# Five Constituents in *Psoralea corylifolia* L. Attenuate Palmitic Acid-Induced Hepatocyte Injury *via* Inhibiting the Protein Kinase C-α/Nicotinamide-Adenine Dinucleotide Phosphate Oxidase Pathway

**DOI:** 10.3389/fphar.2019.01589

**Published:** 2020-01-28

**Authors:** Lishan Zhou, Jianqiao Tang, Xuan Yang, Hui Dong, Xiaoli Xiong, Juan Huang, Linli Zhang, Huan Qin, Suqi Yan

**Affiliations:** ^1^ Department of Integrated Traditional Chinese and Western Medicine, Wuhan Children’s Hospital (Wuhan Maternal and Child Healthcare Hospital), Tongji Medical College, Huazhong University of Science and Technology, Wuhan, China; ^2^ Department of Discipline Inspection and Supervision, Wuhan Children’s Hospital (Wuhan Maternal and Child Healthcare Hospital), Tongji Medical College, Huazhong University of Science and Technology, Wuhan, China; ^3^ Institute of Integrated Traditional Chinese and Western Medicine, Tongji Hospital, Tongji Medical College, Huazhong University of Science and Technology, Wuhan, China; ^4^ Department of Pathology, Wuhan Children’s Hospital (Wuhan Maternal and Child Healthcare Hospital), Tongji Medical College, Huazhong University of Science and Technology, Wuhan, China; ^5^ Hubei University of Chinese Medicine, Wuhan, China; ^6^ Laboratory, Wuhan Children’s Hospital (Wuhan Maternal and Child Healthcare Hospital), Tongji Medical College, Huazhong University of Science and Technology, Wuhan, China

**Keywords:** *Psoralea corylifolia* L., compounds, primary hepatocytes, nonalcoholic fatty liver disease, protein kinase C-α/nicotinamide-adenine dinucleotide phosphate oxidase signaling pathway

## Abstract

*Psoralea corylifolia* L. (PC) is a traditional Chinese herb used to treat yang deficiency of the spleen and kidney in pediatric disease. Our previous studies have found that PC can alleviate the liver oxidative stress of juvenile mice with nonalcoholic steatohepatitis (NASH), and its mechanism is related to the inhibition of the protein kinase C-α (PKC-α)/nicotinamide-adenine dinucleotide phosphate oxidase (NOX) signaling pathway. The aim of this study was to confirm the aforementioned drug target *in vitro* and to conduct preliminary screening for some effective compounds of PC on the treatment of NASH. A primary hepatocyte model of non alcoholic fatty liver disease was established by palmitic acid. The existence of *Psoralen*, *Isopsoralen*, *Neobavaisoflavone*, *Isobavachalcone*, and *Bakuchiol* were identified by ultra-performance liquid chromatography. Then, five PC compounds were administered. Intracellular triglyceride and total cholesterol content, the cell supernatant alanine aminotransferase and aspartate aminotransferase, and hepatocellular superoxide anion were examined. The changes of PKC-α/NOX signaling pathways in hepatocytes were also determined. Furthermore, PKC-α activator phorbol 12-myristate 13-acetate was administered for 4 h before Psoralen intervention was conducted again to detect the changes of PKC-α/NOX signaling pathways. Our data demonstrated that Psoralen, Isopsoralen, and Isobavachalcone decreased intracellular content of triglyceride while all five PC compounds improved hepatocellular total cholesterol accumulation and hepatocyte damage in palmitic acid-induced primary hepatocyte model of non alcoholic fatty liver disease. All five PC compounds could also reduce hepatocytic superoxide anion levels, nicotinamide-adenine dinucleotide phosphate/reduced nicotinamide-adenine dinucleotide phosphate ratio, NOX activity as well as p47^phox^ protein expression and PKCα activation in hepatocytes. Psoralen exhibited the best efficacy but the effectiveness was lost when pre-stimulated by phorbol 12-myristate 13-acetate. The results suggest that Psoralen, Isopsoralen, and Isobavachalcone could improve hepatocyte steatosis; five PC compounds could ameliorate hepatocyte injury, relieve oxidative stress, and downregulate the PKC-α/NOX signaling pathway of hepatocytes. In addition, Psoralen exhibits the best efficacy and a prospective PKC-α inhibitor pharmaceutical activity.

## Introduction

Nonalcoholic fatty liver disease (NAFLD) is the most prominent cause of chronic liver disease worldwide (Younossi et al., 2019). Because 25% of the world’s population is currently thought to have NAFLD, this epidemic has become a severe public health problem (Younossi et al., 2016; Doycheva et al., 2017). NAFLD may result in adverse outcomes of liver diseases (liver cirrhosis, hepatocellular carcinoma) and cardiovascular diseases. Nonalcoholic steatohepatitis (NASH), a severe form of NAFLD, induces a significant increase in all-cause mortality for NAFLD patients (Wu et al., 2016; National Workshop on Fatty Liver and Alcoholic Liver Disease, Chinese Society of Hepatology, Chinese Medical Association. Fatty Liver Expert Committee, Chinese Medical Doctor Association, 2018).

Either a two-hit theory or a multiple hit-process, oxidative stress is one of the key driving forces in the initiation and progression of NAFLD from simple steatosis to NASH (Spahis et al., 2017; Alhasson et al., 2018; Uchida et al., 2018). Oxidative stress refers to a shift in the balance toward increased intracellular reactive oxygen species (ROS) generation, compared to breakdown (Mann et al., 2017). Nicotinamide-adenine dinucleotide phosphate oxidase (NOX) is considered the major cellular ROS source (Loffredo et al., 2016; Long et al., 2017; Rabelo et al., 2018). Its activation has been associated with hepatic injury (Matsumoto et al., 2018; Zheng et al., 2018) and also plays a very plausible role as the starting point of extrahepatic damage, leading to inflammation and fibrosis through activation of Kupffer cells and hepatic stellate cells (HSCs) (Das et al., 2015; Zhou et al., 2018), thus causing a self-perpetuating circle of ROS formation and ROS-mediated damage (Masarone et al., 2018; Zhong and Liu, 2018). Therefore, NOX-specific antioxidant therapies may be a promising intervention to prevent or to treat NASH.

*Psoralea corylifolia* L. (PC), a traditional Chinese herb, a member of Leguminosae plant family, is widely used to treat yang deficiency of the spleen and kidney in both adult and pediatric disease in China. PC plays a beneficial role in multiple diseases, especially cancer and osteoporosis (Xin et al., 2019a). PC also has a wide range of antioxidant activities (Alam et al., 2018). Our previous studies have found that PC can alleviate the liver oxidative stress of juvenile mice with NASH, and its mechanism is related to the inhibition of the PKC-a/NOX signaling pathway (Zhou et al., 2017). Therefore, the aim of this study was to confirm the aforementioned drug target *via* further cell study and to conduct preliminary screening for some effective compounds of PC on the treatment of NASH.

## Materials and Methods

### Reagents

Psoralen (purity: 99.9%; P8399), Isopsoralen (purity: 99%; A0956), Neobavaisoflavone (purity: 99%; SMB00458), Isobavachalcone (purity: 98.8%; SML1450), Bakuchiol (purity: 99.4%; SMB00604), palmitic acid (PA) (P5585) and reduced nicotinamide-adenine dinucleotide phosphate (NADPH) (10107824001) were purchased from Sigma (Saint Louis, MO, USA). Phorbol 12-myristate 13-acetate (PMA) (HY-18739) was purchased from MedChemExpress (Monmouth Junction, NJ, USA). Go6976 (S7119) was purchased from Selleck (Houston, TX, USA). Dulbecco’s modified Eagle medium/F-12 medium (SH30023) was obtained from HyClone (Logan, UT, USA). Fetal bovine serum (10099141) was provided by Gibco (Grand Island, NY, USA). Cell Counting Kit-8 (CCK-8) (C0038) was purchased from Beyotime Institute of Biotechnology (Shanghai, China). Alanine aminotransferase (ALT) (C009-2), aspartate aminotransferase (AST) (C0010-2), triglycerides (TG) (A110-1) and total cholesterol (TC) (A111-1) commercial reagents were purchased from Jiancheng Bioengineering Institute (Nanjing, Jiangsu, China). ROS fluorescence probe-DHE (KGAF019) was obtained from KeyGEN BioTECH (Nanjing, Jiangsu, China). Bicinchoninic acid protein assay kit (AS1086) was offered by ASPEN Biotechnology Co., Ltd. (Wuhan, Hubei, China). Nicotinamide-adenine dinucleotide phosphate (NADP)/NADPH Assay kit (ab65349), lucigenin (ab145310), anti-GAPDH (ab37168), anti-protein kinase C (PKC)-α (ab23513), anti-phosphorylated PKC-α (S657 + Y658) (ab32376), and anti-gp91^phox^ (ab80508) were purchased from Abcam (Hong Kong, China). Anti-p47^phox^ (sc-17845) was obtained from Santa (California, USA). Anti-p47^phox^ (NB100-790) was purchased from NOVUS (Littleton, CO, USA). HRP-goat anti-rabbit (AS-1107), HRP-goat anti-mouse (AS-1106), FITC-goat anti-rabbit (AS-1110) and CY3-donkey anti-goat (AS-1113) were bought by ASPEN Biotechnology Co., Ltd. (Wuhan, Hubei, China).

### Ultra-Performance Liquid Chromatography

Ultra-performance liquid chromatography (UPLC) was performed using a Waters Acquity Ultra Performance LC system (Milford, MA, USA) equipped with a quaternary solvent delivery pump, an autosampler, a thermostated column compartment, a photodiode array detector, and an Empower 3 workstation. The analytes were separated on a Waters Acquity HSST3 column (100 × 2.1 mm, 1.8 um particle). Chromatographic separation was achieved through a 15-min gradient delivery of a mixture of A (acetontrile) and B (1% phosphoric acid aqueous) at a flow rate of 0.3 ml/min and a column temperature of 35°C, using a photodiode array detector (310 nm + full scan).

### Primary Hepatocyte Extraction and Culture

Female C57BL6J mice, aged 24 to 32 days, were chosen for the extraction of hepatocytes. This study was approved by the animal ethics committee of Wuhan Medical & Health Center for Women and Children, Huazhong University of Science and Technology (no. 2014010). A two-step collagenase digestion method was used and cultivated following the steps published by our research group (Xiong et al., 2019). Extracted hepatocytes were cultured in Dulbecco’s modified Eagle medium/F-12 medium supplemented with 10% fetal bovine serum, 100 units/ml penicillin, and 100 mg/ml streptomycin in 5% CO_2_/95% air at 37°C. Immunofluorescence was used to detect CK-18 protein in cells to identify whether the extracted cells were mouse primary hepatocytes (Banaudha et al., 2010).

### Primary Hepatocyte Model of Nonalcoholic Fatty Liver Disease Establishment

Hepatocytes were seeded into 96-well plates and cultured in an incubator overnight. Then, the medium was replaced by medium containing PA at the concentration gradient (0, 0.01, 0.02, 0.05, 0.08, 0.1 mmol/l) for 24 h. The viability of hepatocytes in wells containing different concentrations of PA was assessed by CCK-8 assay. Next, hepatocytes were seeded into 6-well plates and cultured in an incubator overnight. Then, the medium was replaced by medium containing PA at the same concentration gradient for 24 h. The cells were collected, and intracellular content of TG was determined using commercial reagent (GPO-PAP method). The screening concentration of PA for model establishment was 0.02 mmol/l (minimal cytotoxicity with significantly increased intracellular TG content).

### Cytotoxic Effect of *Psoralea corylifolia* L. Chemical Compounds

Hepatocytes were seeded into 96-well plates and cultured in an incubator overnight. Then, the medium was replaced by medium containing 0.02 mmol/l of PA with 1 of each PC chemical compounds (Psoralen, Isopsoralen, Neobavaisoflavone, Isobavachalcone or Bakuchiol) for 24 h. The concentration gradient of all the PC chemical compounds was in turn: 0, 0.2, 2, 20, 50, and 100 μg/ml. The cytotoxic effects of Psoralen, Isopsoralen, Neobavaisoflavone, Isobavachalcone, and Bakuchiol were evaluated by the CCK-8 assay. The screening concentration of all five PC chemical compounds was 0.2 μg/ml (less cytotoxicity with maximum concentration). Primary hepatocyte inhibition ratio of five PC chemical compounds under the selected concentration is listed in **Table 1**.

**Table 1:** Primary hepatocyte inhibition ratio of five PC chemical compounds (n=5).

Item	Selected concentration (μg/ml)	Inhibition ratio (%)
**Psoralen**	0.2	7.46±0.54
**Isopsoralen**	0.2	8.91±1.09
**Neobavaisoflavone**	0.2	9.93±0.81
**Isobavachalcone**	0.2	9.24±1.80
**Bakuchiol**	0.2	9.16±1.49

### Cellular Grouping and Intervention

Groups were divided into control group (A), model group (B), positive control group (C), Psoralen group (D), Isopsoralen group (E), Neobavaisoflavone group (F), Isobavachalcone group (G), and Bakuchiol group (H). Hepatocytes in groups B to H were modeled and treated for 24 h, respectively. Then, different interventions were given as follows: group B (0.02 mmol/l PA); group C (0.02 mmol/l PA + 1 μmol/l Go6976: PKC-α inhibitor); group D (0.02 mmol/l PA + 0.2 μg/ml Psoralen); group E (0.02 mmol/l PA + 0.2 μg/ml Isopsoralen); group F (0.02 mmol/l PA + 0.2μg/ml Neobavaisoflavone); group G (0.02 mmol/l PA + 0.2 μg/ml Isobavachalcone); and group H (0.02 mmol/l PA + 0.2 μg/ml Bakuchiol). The PC chemical compound with the best therapeutic effect (Psoralen) was chosen for further grouping experiments. Model hepatocytes were pre-stimulated for 4 h with activator of PKC-α (100 nmol/l PMA) before PC chemical compound intervention. Groups were divided into control group, model group (0.02 mmol/l PA), Psoralen group (0.02 mmol/l PA + 0.2 μg/ml Psoralen), and Psoralen/activator group (0.02 mmol/l PA + 100 nmol/l PMA + 0.2 μg/ml Psoralen).

### Oil Red O Staining

Hepatocytes were seeded into 6-well plates. Cellular model establishment and intervention abiding by the method described in “item 2.4” and “item 2.6.” After treatment, the cells were washed 3 times with phosphate-buffered saline (PBS) and immobilized with 4% paraformaldehyde for 15 min. After three rinses with distilled water, the cells were dyed with Oil red O at 37°C for 15 min and soon afterward decolorized in 60% isopropanol for 5 s. After 2 rinses with distilled water, the cells were then stained with hematoxylin for 30 s. After washing and the observation of the lipid droplets in hepatocytes, pictures were taken under optical microscope. Five high-power fields were counted, and the stained cell count in 1,000 cells of each high-power field was compared.

### Biochemical Assessment

Hepatocytes were seeded into 6-well plates. Cellular model establishment and intervention followed the method described in “item 2.4” and “item 2.6.” After treatment, the cells and cell supernatant in each group were collected. The cell supernatant level of ALT/AST, and intracellular content of TG/TC were measured using commercial reagents.

### Measurement of Superoxide Anion Levels

For the measurement of ROS, dihydroethidium (DHE), an oxidant-sensitive probe, is widely used. Ethidium and 2-hydroxyethidium, two products of DHE oxidation, bind to the nuclear DNA, forming a strong red fluorescent complex (Zhou et al., 2013; Zhou et al., 2018). Hepatocytes were seeded into 6-well plates. Cellular model establishment and intervention followed the method described in “item 2.4” and “item 2.6.” After treatment, the cells were incubated with DHE (5 mmol/l) in a dark container at 37°C for 30 min, and then nuclei were counterstained with 4’,6-diamidino-2-phenylindole (DAPI) for 10 min. Staining was observed under an inverted microscope (IX51, Olympus, Japan).

### Measurement of Nicotinamide-Adenine Dinucleotide Phosphate/Reduced Nicotinamide-Adenine Dinucleotide Phosphate Ratio

NADPH is essential for redox homeostasis, the exhaustion of NADPH resulted in oxidative stress induction (Liu et al., 2018). Hepatocytes were seeded into 6-well plates. Cellular model establishment and intervention followed the method described in “item 2.4” and “item 2.6.” Hepatocyte NADP/NADPH ratio was measured using an NADP/NADPH Assay kit (Colorimetric) according to the manufacturer’s instructions.

### Measurement of Nicotinamide-Adenine Dinucleotide Phosphate Oxidase Activity

A lucigenin chemiluminescence assay in the presence of NADPH as the substrate was used for measurement of NOX activity (Sung et al., 2017). Hepatocytes were seeded into 6-well plates. Cellular model establishment and intervention followed the method described in “item 2.4” and “item 2.6.” The treated cells were collected, washed with ice PBS twice and then added ice Jude Krebs buffer (119 mmol/l NaCl, 20 mmol/l Hepes, 4.6 mmol/l KCl, 1 mmol/l MgSO_4_, 0.15 mmol/l Na_2_HPO_4_2·H_2_O, 136.09 mmol/l KH_2_PO_4_, 84.01 mmol/l NaHCO_3_, 147mmol/l CaCl2, and 180.2 mmol/l glucose) containing proteinase inhibitor cocktail for lysis cell. The protein concentration was quantified using a bicinchoninic acid protein assay kit. Hepatocyte protein extraction (40 μg), lucigenin (10 μmol/l) and NADPH (100 μmol/l) were added into the 96-well plates to start the reaction. One value per minute was measured with a chemiluminescent instrument for a total of 10 min. Enzyme activity is expressed as the relative light unit of protein per milligram per minute.

### Western Blot Analysis

After centrifugation and determination of total protein concentrations, hepatocyte protein extraction (40 μg) was mixed with sample buffer and boiled for 5 min. According to protein molecular weight, 8 or 10% sodium dodecyl sulfate polyacrylamide gel electrophoresis gel was chosen (120 volts, 90 min). Separated proteins on the gel were transferred to 0.45 μm polyvinylidene fluoride membranes (activated with methanol before use for 3 min). The membranes were then blocked with 5% fat-free dry milk in Tris-buffered saline with Tween (TBST) or 0.5% bovine serum albumin at room temperature for 1 h, followed by incubation with antibodies (**Table 2**) at 4°C overnight. The next day, after washing with TBST three times (5 min per time), the membranes were incubated with HRP-goat anti-rabbit/mouse antibody diluted 1:10,000 at room temperature for 30 min. After washing with TBST four times (5 min per time), immunoreactive proteins were detected using the chemiluminescence method (LiDE110, Canon, Japan). Finally, band densities were determined by AlphaEaseFC software and quantified as the ratio between the optical density (OD) value of the target band and that of GAPDH.

**Table 2:** First antibody details of Western blot analysis.

Antibody	Species	Manufacturer	Dilution ratio
**GAPDH**	Rabbit	Abcam	1:10,000
**p47^phox^**	Mouse	Santa	1:500
**Phosphorylated PKC-α** (S657 + Y658)	Rabbit	Abcam	1:500
**PKC-α**	Rabbit	Abcam	1:500

GAPDH, glyceraldehyde-3-phosphate dehydrogenase; PKC-α, protein kinase C -α.

### Immunofluorescence

After completion of the treatments of the aforementioned further grouping experiments section, cells attached on coverslips were fixed in 4% paraformaldehyde for 30 min. Then, they were incubated with membrane-breaking liquid at room temperature for 10 min and with 3% H_2_O_2_ in the dark for 20 min, followed by incubation with primary antibodies (**Table 3**) at 4°C overnight and secondary antibody (**Table 3**) at 37°C for 50 min. After that, they were stained with DAPI at room temperature for 5 min. Staining was viewed under a laser scanning confocal microscope (LSM880, Zeiss, Germany).

**Table 3:** Antibody details of Immunofluorescence.

Antibody	Species	Manufacturer	Dilution ratio
**p47^phox^** (primary)	Goat	Novus	1:50
**gp91^phox^** (primary)	Rabbit	Abcam	1:50
**CY3- Donkey anti Goat** (secondary)	╱	Aspen	1:50
**FITC- Goat anti Rabbit** (secondary)	╱	Aspen	1:50

### Statistical Analysis

All outcomes represent at least six independent experiments. All of the results were analyzed using SPSS 19.0 statistical software. Statistical significance was assessed by one-way analysis of variance following Kolmogorov–Smirnov normality. With homogeneity of variances, significance between different groups was determined by the least standard deviation test; alternatively, the Games-Howell test was used. A probability of less than 0.05 was considered to be statistically significant.

## Results

### Ultra-Performance Liquid Chromatography Profile of *Psoralea corylifolia* L. Concentrated Granules

**Figure 1:**
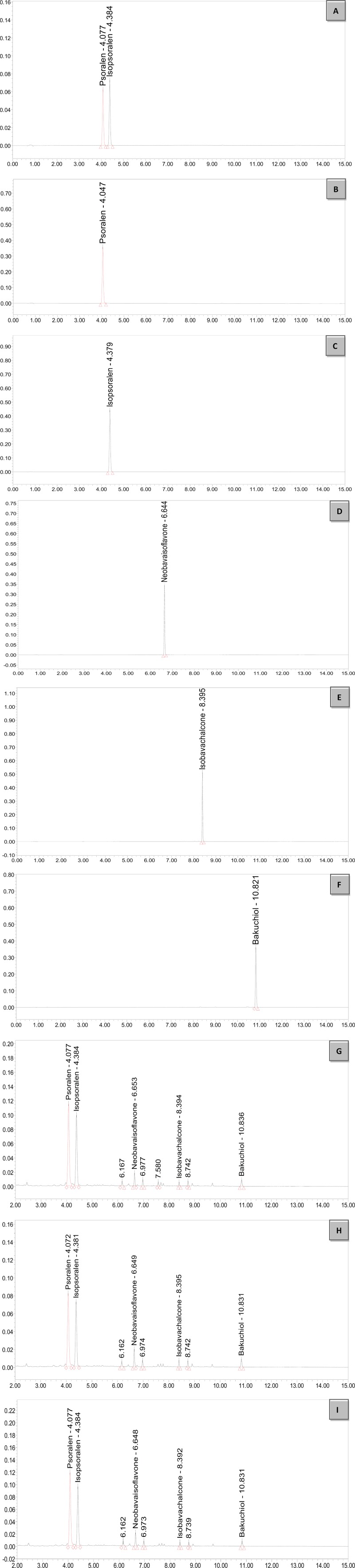
UPLC chromatogram of PC granules from three batches and reference standards. There were three batches of PC granules supplied by Sanjiu Medical and Pharmaceutical Co., Ltd. (Shenzhen, China) (lot: 10601003S, 1712001S, and 1706003S). Units of abscissa: AU; units of ordinates: min. **(A)**: Standard with Psoralen and Isopsoralen; **(B**–**F)**: Standard with Psoralen, Isopsoralen, Neobavaisoflavone, Isobavachalcone, and Bakuchiol; **(G**–**I)**: Three batches of PC granules samples. UPLC, Ultraperformance liquid chromatography.

The major active chemical constituents of PC are coumarins, flavonoids, and meroterpenes. According to Chinese Pharmacopoeia for the criterion of PC, coumarins: Psoralen and Isopsoralen were selected as markers for assessing the quality of the herb and some apropos preparations. UPLC of PC granules from three batches are shown in **Figure 1** and **Table 4**. The result showed that the two main peaks were detected and identified by comparing the retention time with reference standards. Psoralen assay in three batches of PC granules were 5.682, 4.219, and 5.744 mg/g, respectively. Isopsoralen assay in three batches of PC granules were 4.211, 3.202, and 4.013 mg/g, respectively. The result verified that PC-concentrated granules prepared from Chinese traditional medicinal PC and the three batch granules of testing data adhere to the Chinese Pharmacopoeia standard, as qualified samples can be used in the experiment. Moreover, Neobavaisoflavone, Isobavachalcone, and Bakuchiol can be detected in the granules consistent with the peak time of the respective standards although they are low in content. Neobavaisoflavone assay in three batches of PC granules were 0.879, 0.985, and 1.015 mg/g, respectively; Isobavachalcone assay in three batches of PC granules were 0.176, 0.238, and 0.189 mg/g, respectively; Bakuchiol assay in three batches of PC granules were 2.649, 2.677, and 2.367 mg/g, respectively.

**Table 4:** Contents of five PC chemical compounds in three batches of PC granules.

Item	1601003S(mg/g)	1712001S(mg/g)	1706003S(mg/g)
**Psoralen**	5.682	4.219	5.744
**Isopsoralen**	4.211	3.202	4.013
**Neobavaisoflavone**	0.879	0.985	1.015
**Isobavachalcone**	0.176	0.238	0.189
**Bakuchiol**	2.649	2.677	2.367

### Effects of *Psoralea corylifolia* L. Chemical Compounds on Palmitic Acid-Induced Hepatocyte Steatosis and Damage

#### Intracellular Content of Triglycerides and Total Cholesterol

**Figure 2:**
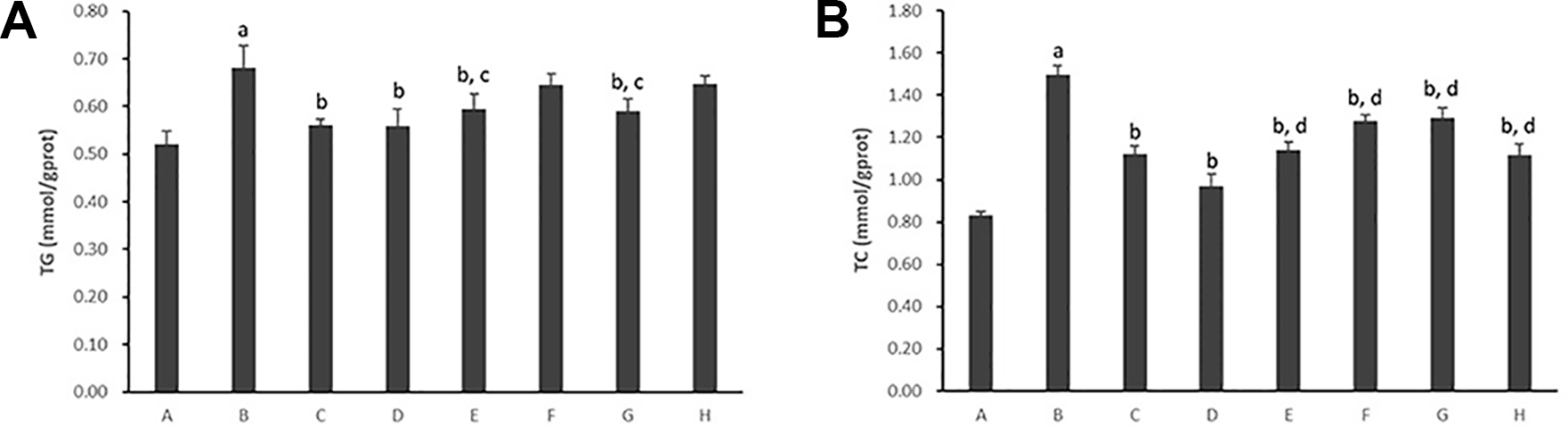
Effects of PC chemical compounds on PA-induced hepatocyte steatosis. Values are mean ± SD (n=6). **(A)** Intracellular content of TG. **(B)** Intracellular content of TC. ^a^ *p* < 0.01 versus group A; ^b^ *p* < 0.01 versus group B; ^c^ *p* < 0.05, ^d^ *p*<0.01 versus group D. Group A (control); group B (0.02 mmol/L PA); group C (0.02 mmol/ L PA+1 μmol/LGo6976); group D (0.02 mmol/L PA+0.2 μg/ml Psoralen); group E (0.02 mmol/L PA +0.2 μg/ml Isopsoralen); group F (0.02 mmol/L PA+ 0.2 μg/ml Neobavaisoflavone); group G (0.02 mmol/L PA+ 0.2 μg/ml Isobavachalcone); group H (0.02 mmol/L PA +0.2 μg/ml Bakuchiol). TG, triglycerides; TC, total cholesterol.

**Figure 3:**
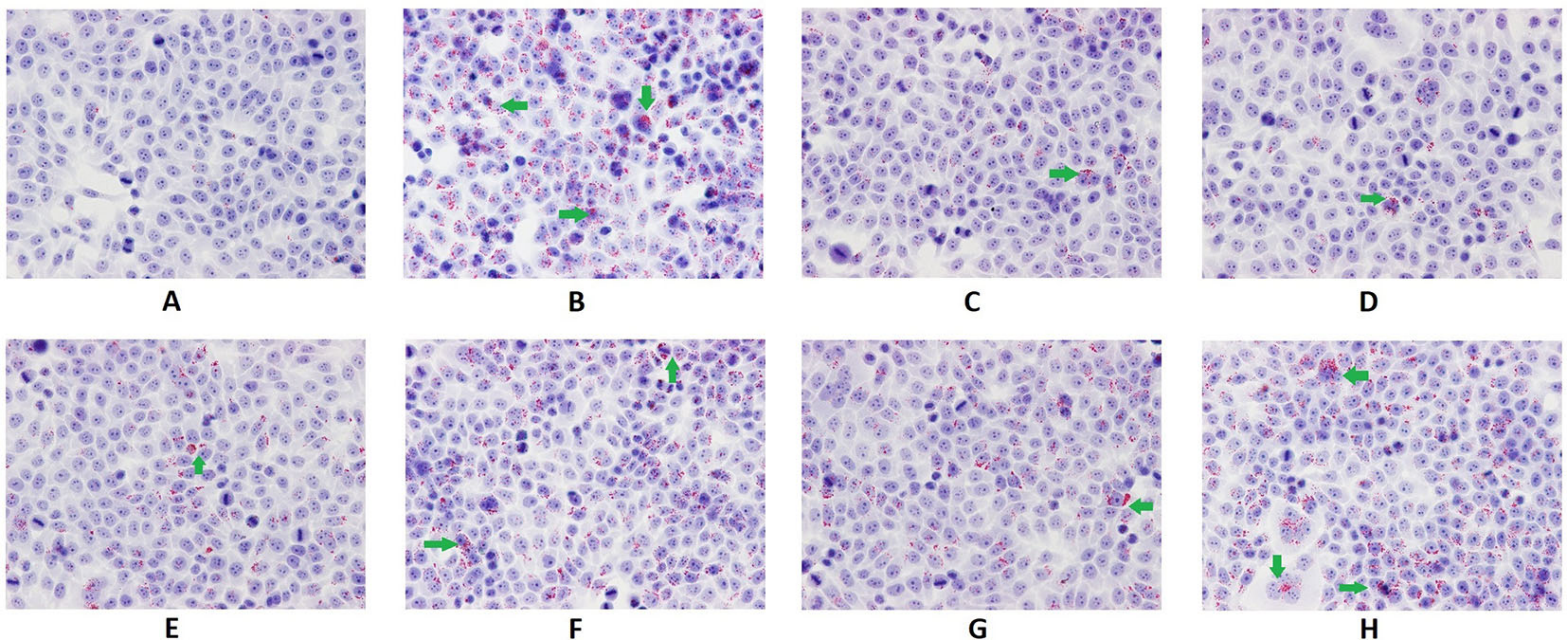
Oil red O staining of hepatocytes in different groups (x400). The green arrows showed spherical lipid droplets of varying sizes in the cytoplasm of steatotic cells, which were red or orange. Group **(A)** (control); group **(B)** (0.02 mmol/L PA); group **(C)** (0.02 mmol/L PA + 1 μmol/L Go6976); group **(D)** (0.02 mmol/L PA + 0.2 μg/ml Psoralen); group **(E)** (0.02 mmol/L PA + 0.2 μg/ml Isopsoralen); group **(F)** (0.02 mmol/L PA + 0.2 μg/ml Neobavaisoflavone); group **(G)** (0.02 mmol/L PA + 0.2 μg/ml Isobavachalcone); group **(H)** (0.02 mmol/L PA + 0.2 μg/ml Bakuchiol).

As shown in **Figure 2**, the intracellular content of TG and TC in hepatocytes cultured with PA markedly increased (*p* < 0.01). The TG contents decreased significantly with the treatment of Psoralen, Isopsoralen, and Isobavachalcone as well as Go6976 (*p* < 0.01). As shown in **Figure 3**, cellular lipid droplets in those treatment groups were diminished compared to the model group. However, the TC content decreased significantly with the treatment of all five PC chemical compounds and Go6976 (*p* < 0.01). Furthermore, a difference in cellular TG/TC content was identified between Psoralen and other PC chemical compounds, indicating a better improvement in TG/TC accumulation (*p* < 0.05, *p* < 0.01).

#### Cell Supernatant Level of Alanine Aminotransferase and Aspartate Aminotransferase

**Figure 4:**
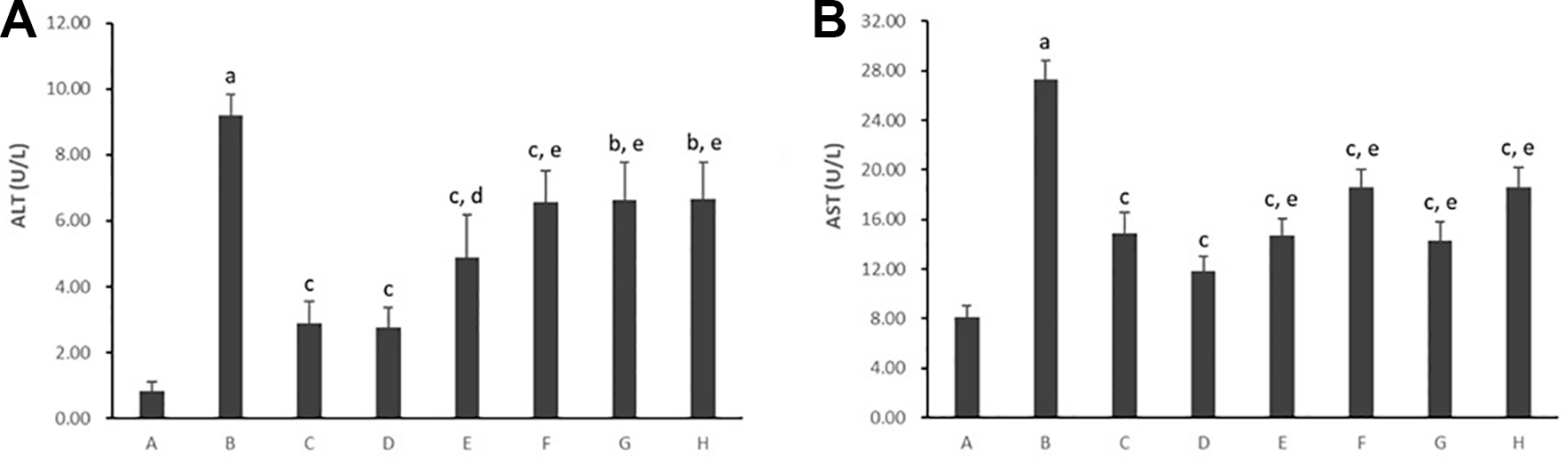
Effects of PC chemical compounds on PA-induced hepatocyte damage. Values are mean ± SD (n = 6). **(A)** Cell supernatant level of ALT. **(B)** Cell supernatant level of AST. ^a^ *p* < 0.01 versus group A; ^b^ *p* < 0.05, ^c^ *p* < 0.01 versus group B; ^d^ *p* < 0.05, ^e^ *p* < 0.01 versus group D. Group A (control); group B (0.02 mmol/L PA); group C (0.02 mmol/L PA + 1 μmol/L Go6976); group D (0.02 mmol/L PA + 0.2 μg/ml Psoralen); group E (0.02 mmol/L PA + 0.2 μg/ml Isopsoralen); group F (0.02 mmol/L PA + 0.2 μg/ml Neobavaisoflavone); group G (0.02 mmol/L PA + 0.2 μg/ml Isobavachalcone); group H (0.02 mmol/L PA + 0.2 μg/ml Bakuchiol). ALT, alanine aminotransferase; AST, aspartate aminotransferase.

As shown in **Figure 4**, untreated hepatocytes cultured with PA presented with both higher cell supernatant ALT and AST levels (*p* < 0.01), which released from damaged hepatocytes. However, treatment with all five PC chemical compounds and Go6976 reversed the increase in cell supernatant ALT and AST levels (*p* < 0.05, *p* < 0.01). Psoralen-treated model hepatocytes presented lower cell supernatant ALT/AST levels than other PC chemical compound-treated model hepatocytes (*p* < 0.05, *p* < 0.01).

### *Psoralea corylifolia* L. Chemical Compounds Prevent Palmitic Acid-Induced Hepatocyte Oxidative Stress

**Figure 5:**
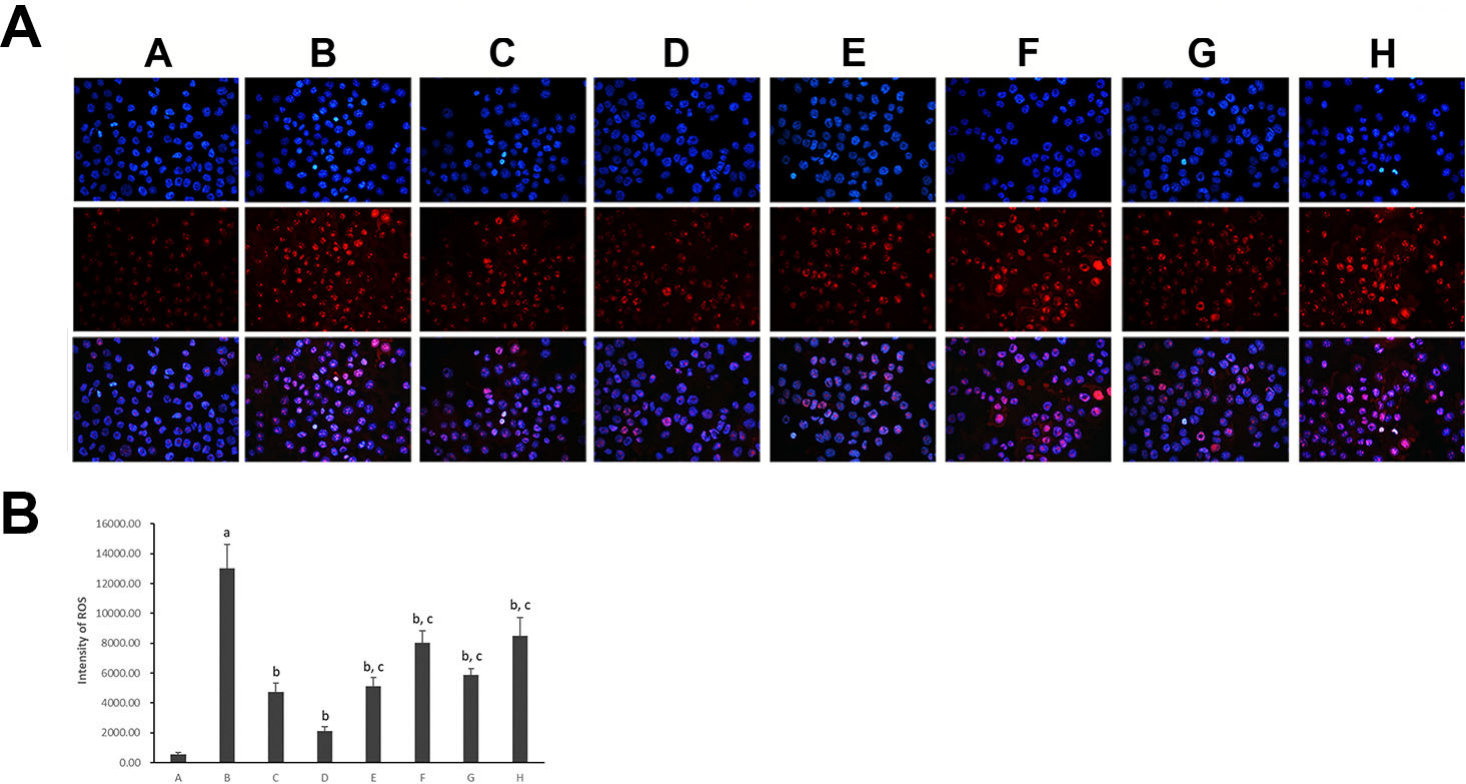
Dihydroethidium staining of hepatocytes in different groups (×400). **(A)** Visualization of the nucleus in hepatocytes using DAPI stains; visualization of ROS in hepatocytes using DHE stains and the superimposed photos of different groups. **(B)** Intensities of DHE staining levels were compared. The fluorescence intensity was obtained by Image-Pro Plus (IPP 6.0) and quantified as integrated option density (IOD) value. Values are mean ± SD (n = 6). ^a^ *p* < 0.01 versus group A; ^b^ *p* < 0.01 versus group B; ^c^ *p* < 0.01 versus group D. Group A (control); group B (0.02 mmol/L PA); group C (0.02 mmol/L PA + 1 μmol/L Go6976); group D (0.02 mmol/L PA + 0.2 μg/ml Psoralen); group E (0.02 mmol/L PA + 0.2 μg/ml Isopsoralen); group F (0.02 mmol/L PA + 0.2 μg/ml Neobavaisoflavone); group G (0.02 mmol/L PA + 0.2 μg/ml Isobavachalcone); group H (0.02 mmol/L PA + 0.2 μg/ml Bakuchiol). DHE: dihydroethidium.

To verify hepatocyte oxidative stress, we estimated the superoxide anion production by DHE staining (**Figure 5**). Compared with the control hepatocytes, a significantly high level of DHE fluorescence was observed in model hepatocytes exposed to PA (*p* < 0.01), indicating increased superoxide anion production. However, treatment with all five PC chemical compounds and Go6976 reduced the level of DHE fluorescence in hepatocytes cultured with PA (*p* < 0.01). Psoralen treatment, compared with other PC chemical compounds, presented the greatest decrease in peroxide anion production in hepatocytes cultured with PA (*p* < 0.01).

### *Psoralea corylifolia* L. Chemical Compounds Inhibit the Liver Protein Kinase C-α/Nicotinamide-Adenine Dinucleotide Phosphate Oxidase Signaling Pathway in Hepatocytes Cultured With Palmitic Acid

#### Hepatocytic Nicotinamide-Adenine Dinucleotide Phosphate/Reduced Nicotinamide-Adenine Dinucleotide Phosphate Ratio

**Figure 6:**
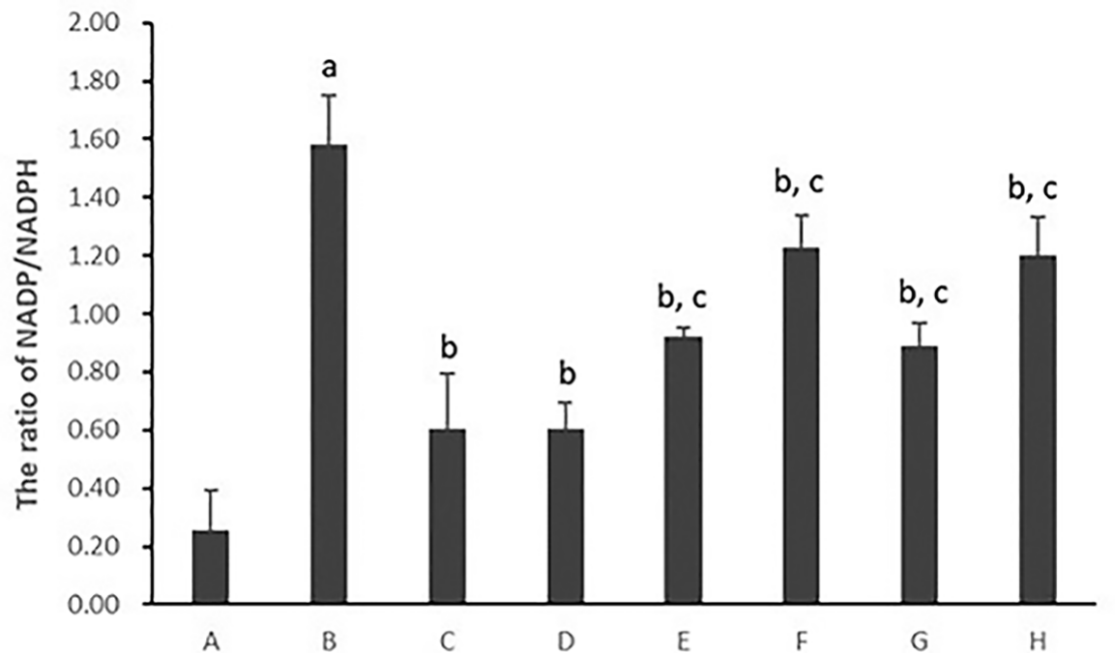
The effect of PC chemical compounds on hepatocytic NADP/NADPH ratio. Values are mean ± SD (n = 6). ^a^ *p* < 0.01 versus group A; ^b^ *p* < 0.01 versus group B; ^c^ *p* < 0.01 versus group D. Group A (control); group B (0.02 mmol/L PA); group C (0.02 mmol/L PA + 1 μmol/L Go6976); group D (0.02 mmol/L PA + 0.2 μg/ml Psoralen); group E (0.02 mmol/L PA + 0.2 μg/ml Isopsoralen); group F (0.02 mmol/L PA + 0.2 μg/ml Neobavaisoflavone); group G (0.02 mmol/L PA + 0.2 μg/ml Isobavachalcone); group H (0.02 mmol/L PA + 0.2 μg/ml Bakuchiol). NADP, nicotinamide-adenine dinucleotide phosphate; NADPH, reduced nicotinamide-adenine dinucleotide phosphate.

As shown in **Figure 6**, the ratio of hepatocytic NADP/NADPH was much higher in hepatocytes cultured with PA (*p* < 0.01). After the treatment with all five PC chemical compounds and Go6976, hepatocytic NADP/NADPH ratio was significantly decreased (*p* < 0.01). Furthermore, Psoralen treatment, compared with other PC chemical compounds, presented the best hepatocytic NADP/NADPH decreasing ratio in hepatocytes cultured with PA (*p* < 0.01).

**Figure 7:**
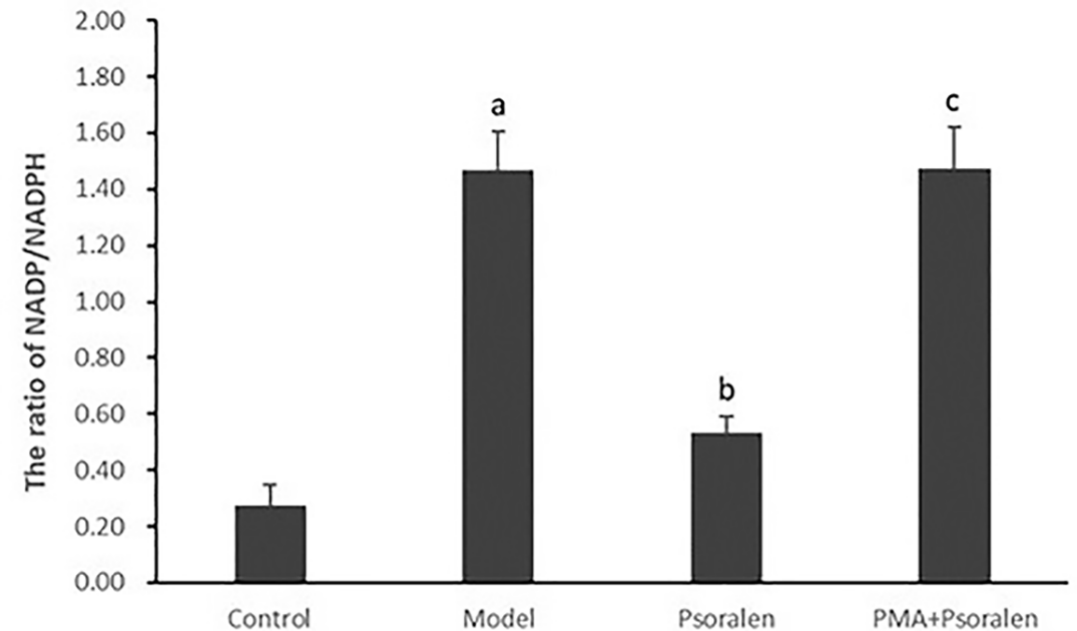
The effect of Psoralen on hepatocytic NADP/NADPH ratio. Values are mean ± SD (n = 6). ^a^ *p* < 0.01 versus control group, ^b^ *p* < 0.01 versus model group, ^c^ *p* < 0.01 versus Psoralen group. Control: Control group; Model: Model group (0.02 mmol/L PA); Psoralen: Psoralen group (0.02 mmol/L PA + 0.2 μg/ml Psoralen); PMA + Psoralen: Psoralen/activator group (0.02 mmol/L PA + 100 nmol/L PMA + 0.2 μg/ml Psoralen). NADP, nicotinamide-adenine dinucleotide phosphate; NADPH, reduced nicotinamide-adenine dinucleotide phosphate.

As shown in **Figure 7**, the ratio of hepatocytic NADP/NADPH was much higher in hepatocytes cultured with PA (*p* < 0.01). After the treatment with Psoralen, hepatocytic NADP/NADPH ratio was significantly decreased (*p* < 0.01). Furthermore, PMA pre-stimulated, compared with Psoralen treatment only, hepatocytic NADP/NADPH ratio was significantly increased (*p* < 0.01).

#### Hepatocytic Nicotinamide-Adenine Dinucleotide Phosphate Oxidase Activity

**Figure 8:**
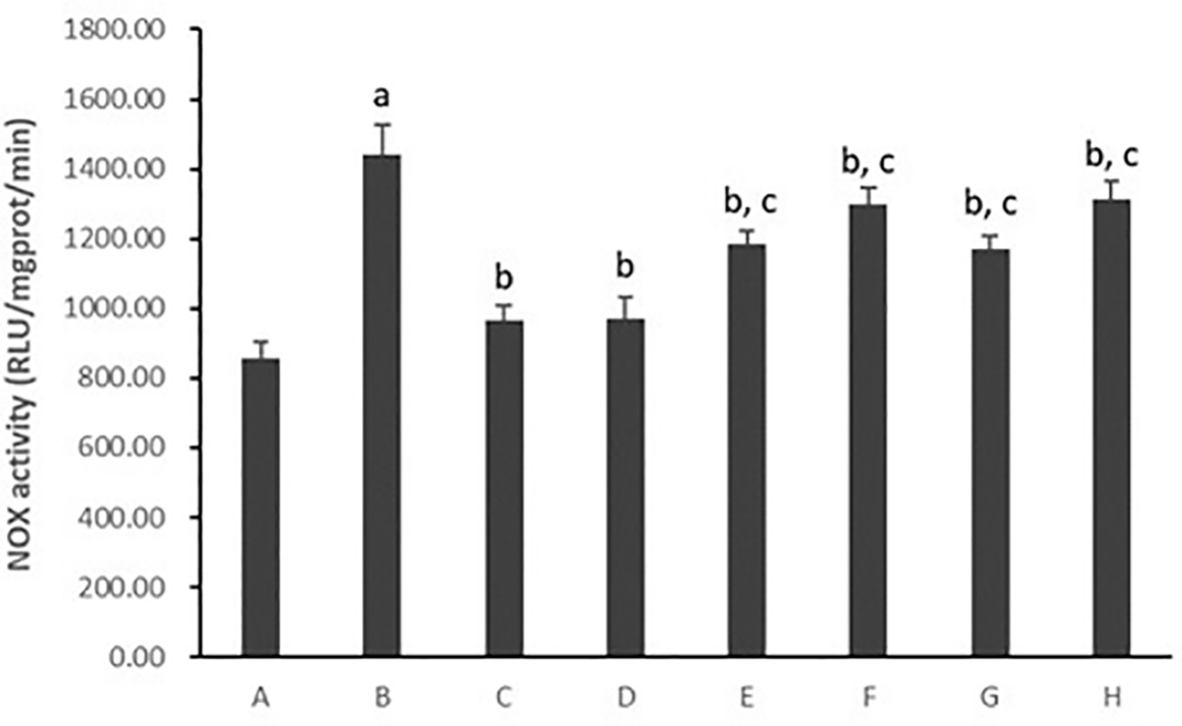
The effect of PC chemical compounds on hepatocytic NOX activity. Values are mean ± SD (n = 6). ^a^ *p* < 0.01 versus group A; ^b^ *p* < 0.01 versus group B; ^c^p < 0.01 versus group D. Group A (control); group B (0.02 mmol/L PA); group C (0.02 mmol/L PA + 1 μmol/L Go6976); group D (0.02 mmol/L PA + 0.2 μg/ml Psoralen); group E (0.02 mmol/L PA + 0.2 μg/ml Isopsoralen); group F (0.02 mmol/L PA + 0.2 μg/ml Neobavaisoflavone); group G (0.02 mmol/L PA + 0.2 μg/ml Isobavachalcone); group H (0.02 mmol/L PA + 0.2 μg/ml Bakuchiol). NOX, Nicotinamide-adenine dinucleotide phosphate oxidase.

**Figure 9:**
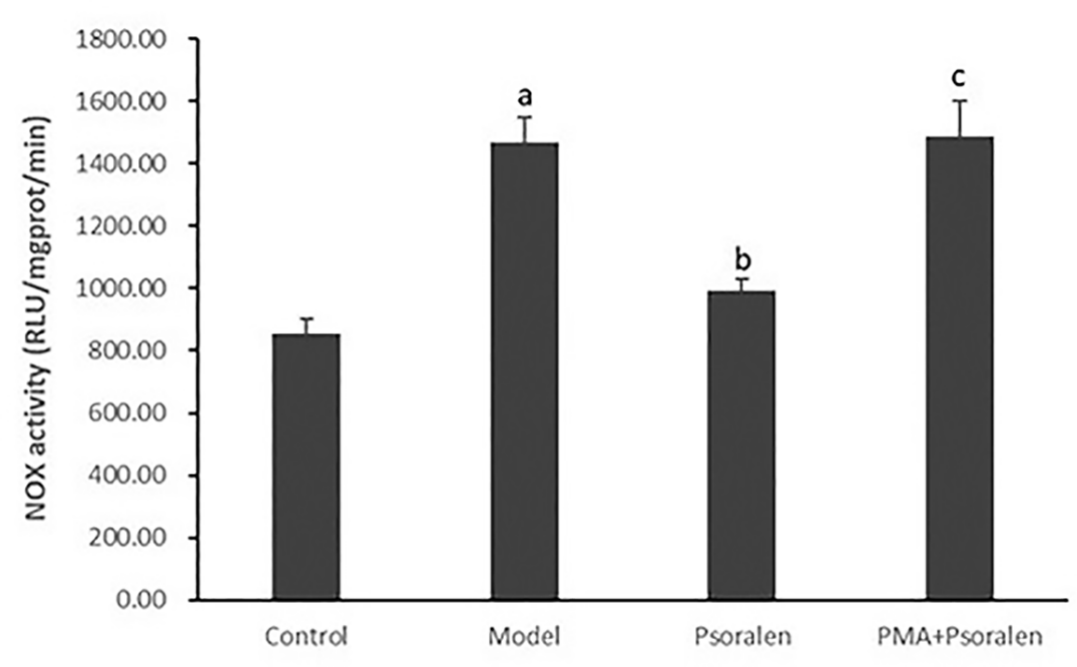
The effect of Psoralen on hepatocytic NOX activity. Values are mean ± SD (n = 6). ^a^ *p* < 0.01 versus control group, ^b^ *p* < 0.01 versus model group, ^c^ *p* < 0.01 versus Psoralen group. Control: Control group; Model: Model group (0.02 mmol/L PA); Psoralen: Psoralen group (0.02 mmol/L PA + 0.2 μg/ml Psoralen); PMA + Psoralen: Psoralen/activator group (0.02 mmol/L PA + 100 nmol/L PMA + 0.2 μg/ml Psoralen). NOX, Nicotinamide-adenine dinucleotide phosphate oxidase.

As shown in **Figures 8** and **9**, hepatocytic NOX activity was evidently enhanced in hepatocytes cultured with PA (*p* < 0.01). After the treatment with all five PC chemical compounds and Go6976, hepatocytic NOX activity was notably decline (*p* < 0.01). Psoralen-treated model hepatocytes presented lower hepatocytic NOX activity than other PC chemical compounds-treated model hepatocytes (p < 0.01). Furthermore, PMA pre-stimulated, compared with Psoralen treatment only, hepatocytic NOX activity was sharply ascend (*p* < 0.01).

**Figure 10:**
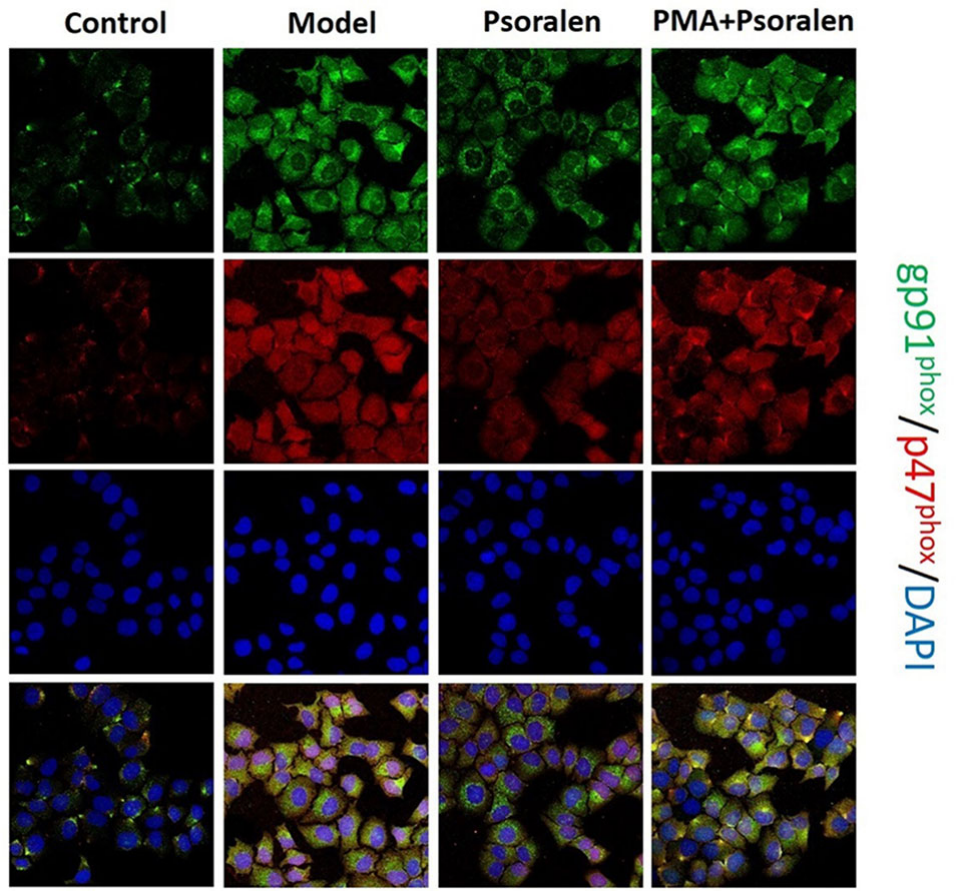
Immunofluorescence of gp91^phox^ and p47^phox^ (×400). gp91^phox^, green; p47^phox^, red; DAPI, blue; co-localization of gp91^phox^/p47^phox^, yellow. Control: Control group; Model: Model group (0.02 mmol/L PA); Psoralen: Psoralen group (0.02 mmol/L PA + 0.2 μg/ml Psoralen); PMA + Psoralen: Psoralen/activator group (0.02 mmol/L PA + 100 nmol/L PMA + 0.2 μg/ml Psoralen).

Besides lucigenin assay, dual-labeled (gp91^phox^-green and p47^phox^-red) immunofluorescence was carried out to see the activation of hepatocytic NOX. Classic NOX (NOX2), expressed on hepatocytes, is composed of several subunits, mainly gp91^phox^ (membrane subunit) and p47^phox^ (cytosolic subunit). NOX2 activation it leads to the alignment of the cytosolic subunit p47^phox^ to the membrane subunit gp91^phox^ (Alhasson et al., 2018). As shown in **Figure 10**, obviously visible gp91^phox^/p47^phox^ co-localization (yellow) events appeared in hepatocytes cultured with PA. After the treatment with Psoralen, the yellow color became significantly lighter. Furthermore, compared with Psoralen treatment only, the yellow color got stronger in the PMA pre-stimulated group.

#### Hepatocytic p47^phox^ Protein Expression

**Figure 11:**
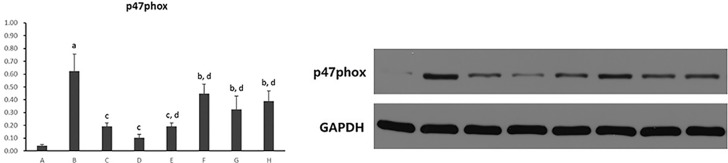
The effect of PC chemical compounds on hepatocytic p47^phox^ protein expression. Values are mean ± SD (n = 6). ^a^ *p* < 0.01 versus control group; ^b^ *p* < 0.05, ^c^ *p* < 0.01 versus group B; ^d^ *p* < 0.01 versus group D. Group A (control); group B (0.02 mmol/L PA); group C (0.02 mmol/L PA + 1 μmol/L Go6976); group D (0.02 mmol/L PA + 0.2 μg/ml Psoralen); group E (0.02 mmol/L PA + 0.2 μg/ml Isopsoralen); group F (0.02 mmol/L PA + 0.2 μg/ml Neobavaisoflavone); group G (0.02 mmol/L PA + 0.2 μg/ml Isobavachalcone); group H (0.02 mmol/L PA + 0.2 μg/ml Bakuchiol).

As shown in **Figure 11**, hepatocytic p47^phox^ protein levels were significantly increased in hepatocytes cultured with PA (*p* < 0.01). However, there was a reduction in the expression of p47^phox^ protein in all five PC chemical compounds and Go6976 groups (*p* < 0.05, *p* < 0.01). Additionally, Psoralen showed the best effect in decreasing p47^phox^ protein expression among PC chemical compounds treatments (*p* < 0.01).

**Figure 12:**
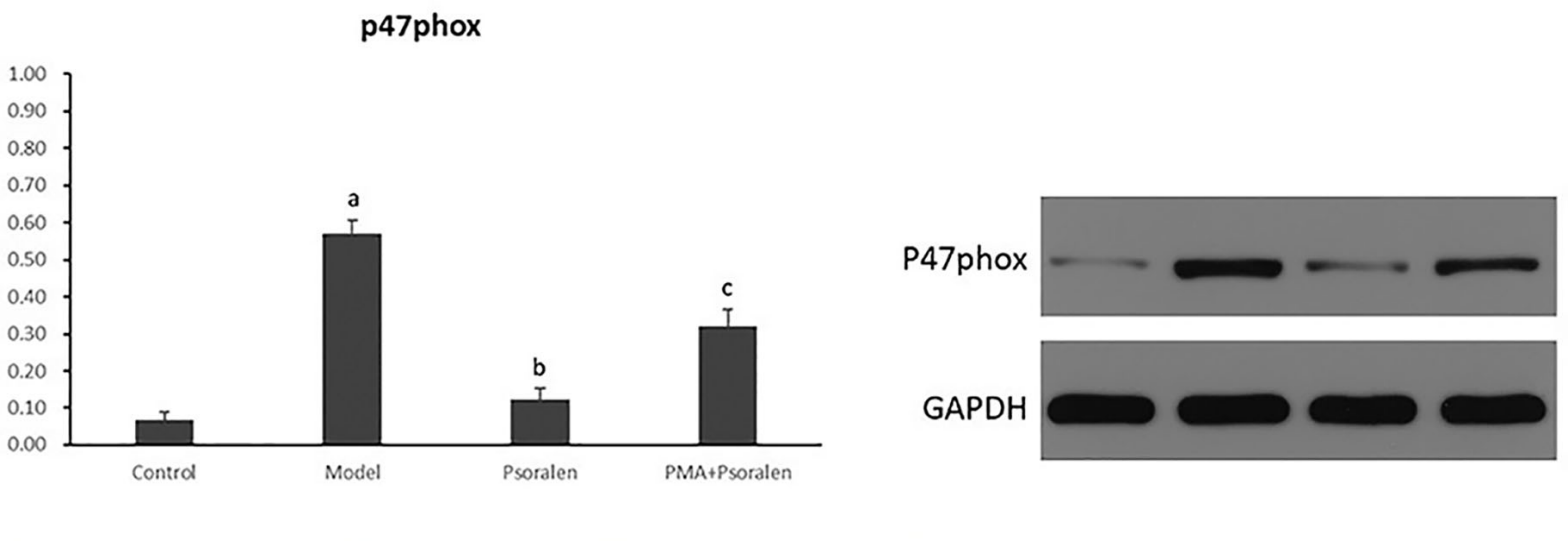
The effect of Psoralen on hepatocytic p47^phox^ protein expression. Values are mean ± SD (n = 6). ^a^p < 0.01 versus control group, ^b^p < 0.01 versus model group, ^c^p < 0.01 versus Psoralen group. Control: Control group; Model: Model group (0.02 mmol/L PA); Psoralen: Psoralen group (0.02 mmol/L PA + 0.2 μg/ml Psoralen); PMA + Psoralen: Psoralen/activator group (0.02 mmol/L PA + 100 nmol/L PMA + 0.2 μg/ml Psoralen).

As shown in **Figure 12**, PA induced overexpression of p47^phox^ in protein (*p* < 0.01) abolished by Psoralen treatment (*p* < 0.01). However, compared with Psoralen treatment only, the expression of p47^phox^ protein was significantly elevated in the PMA pre-stimulated group (*p* < 0.01).

#### Hepatocytic Protein Kinase C-α Activity

**Figure 13:**
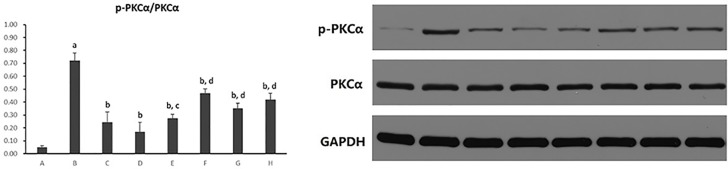
The effect of PC chemical compounds on hepatocytic PKC-α activity. Values are mean ± SD (n = 6). ^a^ *p* < 0.01 versus group A; ^b^ *p* < 0.01 versus group B; ^c^ *p* < 0.05, ^d^ *p* < 0.01 versus group D. Group A (control); group B (0.02 mmol/L PA); group C (0.02 mmol/L PA + 1 μmol/L Go6976); group D (0.02 mmol/L PA + 0.2 μg/ml Psoralen); group E (0.02 mmol/L PA + 0.2 μg/ml Isopsoralen); group F (0.02 mmol/L PA + 0.2 μg/ml Neobavaisoflavone); group G (0.02 mmol/L PA + 0.2 μg/ml Isobavachalcone); group H (0.02 mmol/L PA + 0.2 μg/ml Bakuchiol). p-PKCa, phosphorylated protein kinase C-a; PKCa, protein kinase C-a.

Hepatocytic PKC-α activity was shown as the ratio of phosphorylated PKC-α to PKC-α (p-PKCα/PKCα) protein expression in hepatocytes. As shown in **Figure 13**, it exhibited a marked elevation in p-PKCα/PKCα in hepatocytes exposure to PA (*p* < 0.01). The ratio of p-PKCα/PKCα was significantly reduced after the treatment of all five PC chemical compounds and Go6976 (*p* < 0.01). Moreover, Psoralen intervention showed a reduction of p-PKCα/PKCα when compared with other PC chemical compounds (*p* < 0.05, *p* < 0.01).

**Figure 14:**
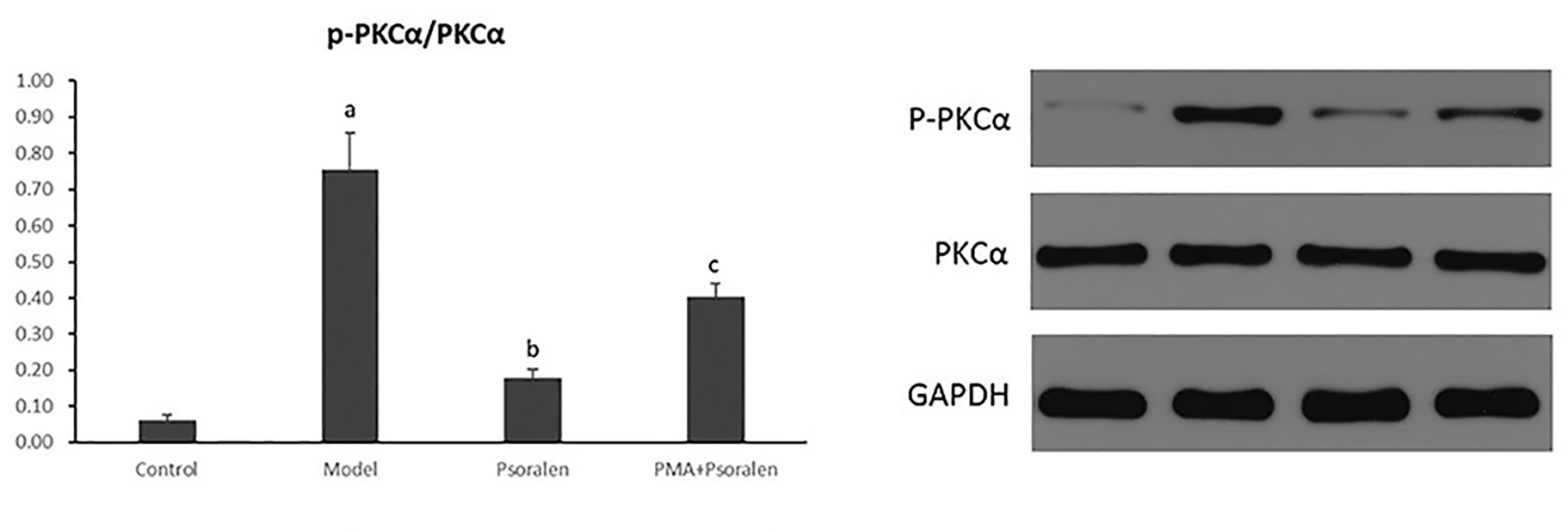
The effect of Psoralen on hepatocytic PKC-α activity. Values are mean ± SD (n = 6). ^a^ *p* < 0.01 versus control group, ^b^ *p* < 0.01 versus model group, ^c^ *p* < 0.01 versus Psoralen group. Control: Control group; Model: Model group (0.02 mmol/L PA); Psoralen: Psoralen group (0.02 mmol/L PA + 0.2 μg/ml Psoralen); PMA + Psoralen: Psoralen/activator group (0.02 mmol/L PA + 100 nmol/L PMA + 0.2 μg/ml Psoralen). p-PKCa, phosphorylated protein kinase C-a; PKCa, protein kinase C-a.

As shown in **Figure 14**, it exhibited a marked elevation in p-PKCα/PKCα in hepatocytes cultured with PA (*p* < 0.01). The ratio of p-PKCα/PKCα was significantly reduced after the treatment of Psoralen (*p* < 0.01). Moreover, PMA pre-stimulated Psoralen intervention showed an elevation of p-PKCα/PKCα when compared with Psoralen treatment only (*p* < 0.01).

## Discussion

The traditional Chinese medicinal herb *Psoralea corylifolia* L. (PC), a hotspot in modern pharmacological research, has many diverse ethnopharmacological and medicinal applications (Alam et al., 2018; Xin et al., 2019a). Thus far, a variety of pharmacological activities of PC have been discovered, including anti-oxidative, antibacterial, anti-inflammatory, anti-depressive, and estrogenic-like effects (Xin et al., 2019b). Our previous studies have reported that PC can treat juvenile NASH mice, attenuate the liver oxidative stress, and inhibit the PKC-a/NOX signaling pathway (Zhou et al., 2017). However, notably, hundreds of compounds have been separated from PC. They belong to various groups, and coumarins, flavonoids, and meroterpenes are more dominant (Zhang et al., 2016). Therefore, based on the extensive chemical and pharmacological research, the anti-oxidative efficacy PC compounds (coumarins: Psoralen and Isopsoralen; flavonoids: Neobavaisoflavone and Isobavachalcone; meroterpenes: Bakuchiol) were selected in this study to do further drug target validation. The result of UPLC verified that PC granules meet the requirements of the Chinese Pharmacopoeia and confirmed the existence of the above five compounds in PC granules.

PA can successfully induce cell steatosis and damage of juvenile mice primary hepatocytes: hepatocytes in the model group showed a large number of lipid droplets, elevated TG and TC contents, and increased the stable intracellular enzymes (ALT, AST) activity of hepatocytes. Psoralen, Isopsoralen, and Isobavachalcone could relieve both intracellular TG and TC contents. Neobavaisoflavone and Bakuchiol could only relieve intracellular TC content, but all five PC compounds could attenuate hepatocytic injury, and both cell supernatant ALT and AST levels were reduced. Moreover, Psoralen was the best in the aforementioned effects than others PC compounds. These results suggest that both two coumarins (Psoralen and Isopsoralen) and one of the flavonoids (Isobavachalcone) of PC compounds can improve PA-induced juvenile mice primary hepatocytic steatosis and damage, and the therapeutic effect of Psoralen is superior.

As for progression of NAFLD/NASH, oxidative stress is the most crucial pathological event during NAFLD development and the hallmark between simple steatosis and NASH manifestation (Spahis et al., 2017), contributing to the aggravation of the liver fibrosis process (Ratziu et al., 2010). Hence, according to our previous researches about anti-oxidative bioactivity of PC (Zhou et al., 2013; Zhou et al., 2017), this study further focused on investigating the changes of oxidative stress situation in different PC compound groups. As in our previous *in vivo* study (Zhou et al., 2017; Zhou et al., 2018), DHE staining was performed to calculate superoxide anion production of hepatocytes. The appearance of superoxide can change DHE into ethidium bromide, which binds to DNA and exhibits red fluorescence in the nucleus (Gracia-Sancho et al., 2008; Vazquez-Chantada et al., 2013). In this study, a massive increase in superoxide anion production was identified in PA-induced model hepatocytes with NASH, and an amelioration effect was shown after all five PC compounds intervention. However, Psoralen treatment, compared with other PC compounds, presented the maximum decreasing of superoxide anion production. These findings further confirm the beneficial effect of all three main categories of PC compounds (coumarins, flavonoids, and meroterpenes) on improving oxidative stress in *in vitro* study.

As mentioned, NOX plays a pivotal role in the development of NASH (Zhou et al., 2018). In the liver, NOX is functionally expressed both in the classic phagocytic form and in the nonphagocytic form. The mammalian NOX family comprises seven isoforms: NOX1, NOX2, NOX3, NOX4, NOX5, DUOX1, and DUOX2. Hepatocytes express mRNAs for NOX2 and regulatory subunit p47^phox^ (Paik et al., 2014). Our previous studies also described that PC was effective in the treatment of NASH in juvenile female mice, and the underlying mechanism might be related to the attenuation of hepatic oxidative stress *via* PKC-α/NOX signaling pathway (Zhou et al., 2017). Thus, the examination of the NADP/NADPH ratio, the NOX activity and the gene expression of its regulatory subunit p47^phox^ and upstream PKC-α were continued in the current study. We found that all the five PC compounds significantly reduced NADP/NADPH ratio, inhibited NOX activity and p47^phox^ protein expression in PA-induced primary hepatocytes with NASH. PKC-α activity was also decreased after intervention of all five PC compounds. Psoralen was superior to others PC compounds in attaining the aforementioned outcomes. These results demonstrated that the inhibition of all three main categories of PC compounds (coumarins, flavonoids, and meroterpenes) on ROS production might be related to the downregulation of the PKC-α/NOX signaling pathway in hepatocytes.

Subsequently, we added activator of PKC-α to pre-stimulate model hepatocytes before Psoralen intervention for further testing the possibility of Psoralen as a candidate PKC-α inhibitor. In this part, we re-evaluated the changes of the NADP/NADPH ratio, the activity of NOX and the gene expression of its regulatory subunit p47^phox^ and upstream PKC-α. Additionally, observation of cytosolic subunit p47^phox^ translocated to the membrane for assembly of the active complex of NOX enzymes. We found that in the PKC-α activator pre-stimulated group vs. Psoralen alone treatment group, the trend of changes of re-estimated items were noticeably reversed, accomplished with the decrease of gp91^phox^/p47^phox^ membrane translocation. The results demonstrated a negative effect on downregulation of the PKC-α/NOX signaling pathway when PKC-α activator and Psoralen were combined.

In summary, we concluded that Psoralen, Isopsoralen, and Isobavachalcone could improve the steatosis of hepatocytes. Five PC compounds (coumarins: Psoralen and Isopsoralen; flavonoids: Neobavaisoflavone and Isobavachalcone; meroterpenes: Bakuchiol) could ameliorate hepatocyte injury, relieve oxidative stress, and downregulate the PKC-α/NOX signaling pathway of hepatocyte. In addition, Psoralen exhibits the best efficacy and a prospective PKC-α inhibitor pharmaceutical activity.

Before the end, we would like to raise two important problems for the research of PC in the further. Firstly, the chemical composition of PC has two-sided effect on liver. Thus, the side of hepatotoxicity should not be ignored. A systematic and comprehensive study of domestic and foreign literatures and information has been made for safety evaluation and risk control measures of PC (Tian et al., 2017). This result suggested that PC may lead to liver damage, but the toxic components and mechanisms of PC have not been clear at present due to its complicated ingredients. Therefore, strictly apply PC in clinic is emphasized (According to Chinese Pharmacopoeia, the upper limit of the dosage for human body is 10 g/d.) and safety related basic and clinical studies are urgently required so as to full exert the efficacy and avoid the PC risk. Secondly, it was reported that copper may play a key role in NAFLD pathogenesis. A tight link between copper homeostasis and lipid metabolism has been well recognized (Feldman et al., 2015; Tarantino et al., 2018). Resent study found that adipocyte-specific Atp7a, a P-type ATPase that transports copper across cell membranes, knockout mice present hepatic steatosis and upregulated peroxisome proliferator activated receptor γ (PPARγ) in gonadal white adipose tissue (Tao et al., 2019). Moreover, copper has a great redox potential and altered copper levels can promote oxidative stress occurrence. New research detected that Bavachinin (BNN), one of the main active ingredients of PC, exhibits anti-tumor activity against non-small cell lung cancer by activating PPARγ induced ROS increasing (Ge et al., 2019). These studies provide a new direction for exploring the protective effect of PC on liver, which may be involved in its impact on copper and related oxidative system.

Finally, we are aware of the limitation of the present study. Lack of lentiviral infection and siRNA-mediated gene silencing, the PKC-α overexpression and downregulation hepatocyte model are missing. Therefore, whether Psoralen directly inhibit PKC-α to be further verify in upregulated and downregulated PKCα expression hepatocyte models.

## Data Availability Statement

All datasets generated for this study are included in the article.

## Ethics Statement

The animal study for the extraction of hepatocytes was reviewed and approved by the animal ethics committee of Wuhan Medical & Health Center for Women and Children, Huazhong University of Science and Technology (No. 2014010).

## Author Contributions

LZho and SY conceived and designed the study. LZho, JT, XY, XX, JH, LZha, and HQ performed the experiments. LZho and HQ wrote the paper. LZho and HD reviewed and edited the manuscript. All authors read and approved the manuscript.

## Funding

This article was supported by the National Natural Science Foundation of China (no. 81403251, 81574024). Medical Training Project for Young and Middle-aged Backbone Talents in Wuhan (Health and Family Planning Commission of Wuhan Municipality [2017] no.51).

## Conflict of Interest

The authors declare that the research was conducted in the absence of any commercial or financial relationships that could be construed as a potential conflict of interest.
